# Gastric Perforation and Pancreatitis Manifesting after an Inadvertent Nissen Fundoplication in a Patient with Superior Mesenteric Artery Syndrome

**DOI:** 10.1155/2009/426162

**Published:** 2009-08-27

**Authors:** Mikael Petrosyan, Joaquin J. Estrada, Stefano Giuliani, Monica Williams, Heather Rosen, Rodney J. Mason

**Affiliations:** Division of Emergency Surgery, Department of Surgery, Keck School of Medicine, University of Southern California Medical Center and Los Angeles County Hospital, Los Angeles, CA 90033, USA

## Abstract

Superior mesenteric artery (SMA) syndrome is an uncommon but well-recognized clinical entity. It can lead to proximal small bowel obstruction and severe morbidity and mortality in lieu of late diagnosis and concomitant existing comorbidities. We report a 54-year-old female, with SMA syndrome which manifested itself after Nissen fundoplication along with two major complications. The diagnosis of SMA was established by clinical symptoms and radiological findings.

## 1. Introduction

Superior mesenteric artery syndrome (SMAS), which is also known as Cast syndrome or Wilkie syndrome, was first described in 1861 by Wilkie [1]. SMAS syndrome is characterized by compression of the third, or transverse, portion of the duodenum against the aorta by the superior mesenteric artery (SMA) secondary to a narrowing of the angle between these two vessels. It leads to chronic, intermittent, acute complete and/or partial duodenal obstruction [2, 3]. The precise incidence of this entity is unknown; however literature suggests that approximately 0.013–0.3% of upper gastrointestinal tract barium studies support a diagnosis of SMAS [4, 5].

## 2. Case Report

A 54-year-old Caucasian female presented to the clinic with epigastric pain and bloating after meals. Seven months ago she underwent a laparoscopic Nissen fundoplication for refractory gastroesophageal reflux disease in her native country. Her reflux symptoms started 2 years ago which included severe heartburn, retrosternal crampy pains as well globus sensation. She was initially treated with PPIs for a period of 4 months with only minimal improvement of her symptoms. She underwent laparoscopic Nissen Fundoplication with unremarkable hospital course and was discharged home in the stable condition on the third postoperative day. Shortly after her surgery she left her country and traveled to Los Angeles, only to find herself in an urgent care clinic few days later where she was evaluated for her daily bloating episodes lasting 2-3 hours, inability to belch, and intermittent nausea. After thorough evaluation, which included laboratory work and abdominal X-ray, it was concluded that her complaints were most likely related to a normal post-Nissen fundopication symptoms. She was reassured and subsequently discharged home. However, she continued to have similar symptoms and several weeks later she represented to a local hospital with severe abdominal pain and fevers of 101 F. Her laboratory work revealed elevated WBC 18 000 cells/*μ*L (normal, 4500–10 500 cells/*μ*L). She had sinus tachycardia with the systolic blood pressure of 90 and abdominal examination consisting of generalized peritonitis. She was taken emergently to the operating room for exploratory laparotomy. Intraoperative findings revealed an acute gastric linear tear on the anterior portion of the body of the stomach, 10 centimeters distal to the intact fundoplication wrap. The gastric tear appeared traumatic in origin without any other gastric pathology. Two-layer primary closure of the defect was performed. She remained stable during her hospital stay and was discharged home on 5th postoperative day tolerating regular diet. Two months after her second surgery, she represented again to the emergency department with severe epigastric pain radiating to the back. She was diagnosed with acute pancreatitis on the basis of her symptoms and laboratory values of amylase and lipase 800 UL (normal 60–180 U/L) and 1200 U/L (normal <190 U/L), respectively. Ultrasound and endoscopic retrograde cholangiopancreatography on the admission revealed no evidence of gallstones or choledocholithiasis. Her pancreatitis responded to conservative treatment and she was subsequently discharged home 6 days after her admission with complete resolution of symptoms.

**Figure 1:**
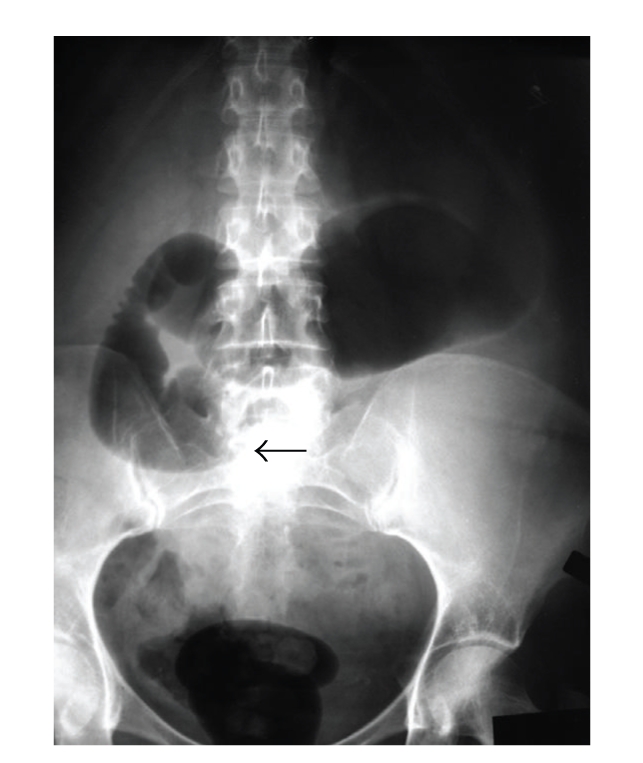
Abdominal X-ray demonstrating abrupt cut off sign at the 3rd portion of duodenum. Dilated stomach and proximal duodenum. Black arrow shows the cut-off sign.

**Figure 2:**
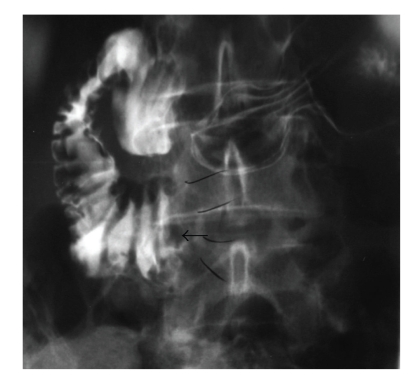
Upper GI demonstrates obstruction at the 3rd portion of the duodenum. Arrow indicates the cut-off sign.

**Figure 3:**
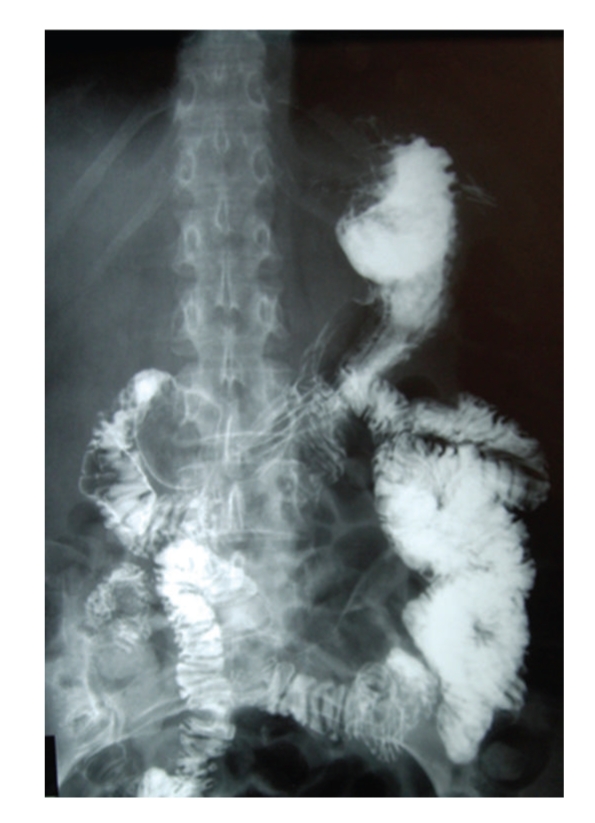
Upper GI shows the relief of obstruction by contrast moving distally, once the patient was placed in prone position.

 During her surgical follow-up visit she continued to complain of postprandial pain with an early satiety and episodic nausea. She appeared weak and malnourished. She noted a 20 lbs weight loss since her initial antireflux procedure. Her abdomen was slightly distended with hyperactive bowel sounds. She had moderate epigastric pain during palpation. She was admitted to the hospital for a work-up of her ongoing symptoms and as well as for poor nutritional status. Her work-up included plain abdominal films, which demonstrated air in a dilated stomach and duodenum with an abrupt vertical cut off and no evidence of air in the small intestine distal to the 3rd portion of the duodenum (Figure 1). Upper Gastrointestinal series obtained subsequently revealed contrast in the dilated duodenum with an abrupt cut off at the level of the superior mesenteric artery pedicle in a supine position (Figure 2). Obstruction was relieved once patient was placed in the prone position (Figure 3). Based on her clinical signs as well as classic radiological findings, a diagnosis of superior mesenteric artery syndrome was made. Patient was taken to the operating room where she underwent duodenojejunostomy. Her postoperative hospital course was uneventful. She was tolerating regular diet and was subsequently discharged home on the 5th postoperative day. Patient remains asymptomatic at one year followup.

## 3. Discussion

Superior mesenteric artery syndrome (SMAS) has been reported as one of the numerous causes of proximal bowel obstruction. Its existence however remains controversial since the presenting symptoms may be similar to other causes of bowel obstruction [2]. Therefore, careful and thorough work-up is required to exclude other causes of bowel obstruction before the diagnosis of SMAS can be made. SMAS has been associated with tumors, peptic ulcer disease, pancreatitis, and intra-abdominal inflammation [3]. Many still question whether SMAS is a true entity while others feel that the syndrome maybe overdiagnozed [4, 5]. The true etiology of this syndrome is related to the anatomical relation of duodenum and superior mesenteric artery (SMA). The disease manifests itself when mechanical compression of duodenum occurs between SMA and its origin, aorta. Various predisposing factors have been described including severe weight loss, prolonged immobilization, pressure and lack of retroperitoneal fat cushion at the SMA, and aortic bufircation [6, 7]. 

We believe that in our patient the precipitating factor was her initial antireflux procedure. The mechanics of Nissen fundoplication with coexisting SMAS in our patient resulted in a closed loop obstruction which led to two subsequent life threatening complications: gastric perforation and pancreatitis. 

Patients with SMAS usually suffer from nonspecific symptoms such as abdominal pain, postprandial fullness, weight loss, bloating, epigastric pain, nausea, and vomiting [7]. Similar symptoms have been described after Nissen fundoplication and therefore led to a misdiagnosis in our case [8]. Moreover, we speculate that her initial reflux symptoms might have been exacerbated or even caused by her proximal duodenal obstruction. Thus, her initial treatment with PPIs was deemed to be a failure, given the inherent mechanics and fluid dynamics of proximal duodenal obstruction. 

 Nonspecific findings of SMAS on a physical examination make the diagnosis even more challenging. Significant complications have been reported in the literature, with fatalities due to severe electrolyte abnormalities, gastric dilatation with subsequent perforation, gastric pneumatosis, portal venous gas, and pancreatitis [9–11]. These complications are all consequences of an increased intraluminal pressure. Although Nissen fundoplication restores the mechanical properties of the stomach cardia and gastroesophageal junction, in our patient, this increased pressure gradient at the cardia together with the duodenal obstruction distally by SMA caused significant increase in the intraluminal pressure both in the stomach and the duodenum. Furthermore, patients with GERD usually swallow more air than normal subjects [12]. This aerophagia can persists for a few month after Nissen Fundoplication and together with decreased ability to belch can lead to gas bloat syndrome. All these factors, including her significant weight loss, might have led to an increased intraluminal pressure with resultant spontaneous gastric perforation. This was due to a closed loop obstruction, which resulted from obstruction proximally by esophageal wrap and distally by SMA. This rare entity has also been described in a patient after laparoscopic Roux-en-Y procedure, most likely from fast and significant weight loss creating a closed loop obstruction [13]. Moreover, pancreatitis in our patient was most likely secondary to abnormal pancreatico-duodenal reflux within the closed loop of the intestine. 

 Symptoms such as bloating, inability to belch, and nausea can be early symptoms of post-Nissen Fundoplication. These symptoms are mostly gastric in origin and are due to mechanical alterations in the cardia of the stomach. If these symptoms persist work-up is warranted. The work-up should include a barium swallow with small bowel follow through and upper endoscopy, therefore evaluating esophageal function, wrap viability, and anatomical visualization of proximal intestine. Moreover, initial work-up of GERD should include Barium swallow, upper endoscopy and 24-hour pH monitoring. If delayed films are obtained after Barium swallow, the proximal duodenal anatomy can be visualized and incidental SMAS can be diagnosed on the initial work-up of GERD. 

 Diagnostic evaluation in suspected SMAS should start with a plain abdominal film. Findings such as abrupt vertical or oblique compression of the third part of the duodenum, dilatation of the second part of duodenum as well as gastric dilation are indicative of this syndrome [14]. To visualize vascular anatomy and the degree of compression of the duodenum, contrast enhanced spiral CT also has been recommended [15, 16]. 

Initial treatment should consist of resuscitation and decompression of the stomach and proximal duodenum with a nasogastric tube. Fast and significant weight loss, which is usually seen in these patients, should be reversed. Surgery is reserved for the patients who fail conservative treatment. Open or laparoscopic duodenojejunostomy is indicated [9].

In conclusion SMAS may often be overlooked as the cause of bowel obstruction. Establishing diagnosis early is very important for fast and effective treatment to improve the patient's condition and prevent subsequent morbidity and mortality associated with this disease entity.
